# Cardiovascular guidelines: separate career may help attenuate controversy

**DOI:** 10.1186/1475-2840-13-66

**Published:** 2014-03-28

**Authors:** Katherine Esposito, Antonio Ceriello, Stefano Genovese, Dario Giugliano

**Affiliations:** 1 Department of Clinical and Experimental Medicine, Second University of Naples, Naples, Italy; 2 Institut d’Investigations Biomèdiques August Pi i Sunyer (IDIBAPS), Barcelona, Spain; 3 Centro de Investigacion Biomèdica en Red de Diabetes y Enfermedades Metabolicas Asociadas (CIBERDEM), Barcelona, Spain; 4 Department of Cardiovascular and Metabolic Diseases, IRCCS Multimedica, Sesto San Giovanni, Milan, Italy; 5 Department of Medical, Surgical, Neurological, Metabolic Sciences and Aging, Second University of Naples, Naples, Italy

## Abstract

The release of recent guidelines for high cholesterol, hypertension and diabetes in the U.S. has been accompanied by great noise and concerns, both in the academic circuits and the lay press. For persons aged 40 to75 years, with LDL cholesterol levels between 70–189 mg/dL and 7.5% or higher estimated 10-year risk, the peril of a global “statinization” has been advocated, predicting a 70% increase of statin use in this otherwise healthy people. A minority of the Eight Joint National Committee panel disagreed with the recommendation to increase the target systolic blood pressure from 140 to 150 mmHg in persons aged 60 years or older without diabetes mellitus or chronic kidney disease. The 2013-American Association of Clinical Endocrinologists algorithm and consensus statement on diabetes has been criticized with particular concerns about transparency, conflicts of interest, group composition, and the abundant use of personal judgment and experience instead of rigorous methodology. Separate careers for experts who collect evidence from persons who write the actual guidelines seems a good opportunity in order to attenuate the noise associated with release of new guidelines, especially those that counter prior practice.

## Introduction

“Patients and the public benefit when physicians and researchers collaborate with pharmaceutical, medical device, and biotechnology companies to develop products that benefit individual and public health. At the same time, concerns are growing that wide-ranging financial ties to industry may unduly influence professional judgments involving the primary interests and goals of medicine. Such conflicts of interest threaten the integrity of scientific investigations, the objectivity of professional education, the quality of patient care, and the public’s trust in medicine” [1].

On 13 November 2013, from the columns of the New York Times (http://www.nytimes.com/2013/11/14/opinion/dont-give-more-patients-statins.html?_r=0), Abramson and Redberg exhorted healthy Americans to focus on the real factors that undeniably reduce the risk of heart disease (healthy diets, exercise, avoiding smoking) and not on statin prevention, as suggested by the new cholesterol guidelines from the American College of Cardiology (ACC) and the American Heart Association (AHA) [2]. The new, long-awaited and much anticipated, hypertension guidelines from the Eighth Joint National Committee (JNC8) [3] are even criticized by members of the very panel that developed them [4]. The validity of the 2013-American Association of Clinical Endocrinologists (AACE) algorithm and consensus statement on diabetes [5] has fiercely been questioned [6]. Are all these concerns intellectually and scientifically justified, or are they related to the evidence that guidelines per se generate controversy, given the complexity of the task and the limitations of available evidence?

## The ACC/AHA cholesterol guidelines

The ACC/AHA guidelines on the treatment of blood cholesterol [2] recommends moderate- to high-intensity statin therapy for primary prevention for the following groups (class I recommendations): (1) persons with low-density lipoprotein (LDL) cholesterol levels of 190 mg/dL or higher; (2) persons aged 40 to75 years with type 1 or 2 diabetes; or (3) persons aged 40 to 75 years with LDL cholesterol levels between 70 and 189 mg/dL and 7.5% or higher estimated 10-year risk of atherosclerotic cardiovascular disease. The peril of a global “statinization” of the planet for this grey zone of cardiovascular risk has been put forward [7], predicting an increase of healthy people for whom statins are recommended by nearly 70 percent (about 920 million people around the world would be classified in this risk categories), and a cumulative global sales of statins approaching $1 trillion by 2020. Critics also point out that 8 of the 15 panelists of the new cholesterol guidelines had industry ties [8].

## THE JNC8 hypertension guidelines

Only about half of patients with hypertension in the United States actually have an systolic blood pressure (SBP) of 140 mm Hg or less [9]. The Joint National Committee (JNC7) Guidelines [10], released more than a decade ago, concluded that all adult patients with hypertension (regardless of their age) should have their BP reduced to a SBP of lower than 140 mm Hg, with even tighter control in patients with diabetes or renal disease (SBP <130 mmHg). In contrast, the current recommendation [3] raises target SBP goals to 150 mm Hg or lower in those aged 60 years or older, while eliminating the tighter control recommendations in patients with diabetes and renal disease. A minority of the panel disagreed with the recommendation to increase the target SBP from 140 to 150 mmHg in persons aged 60 years or older without diabetes mellitus or chronic kidney disease [4]. Intuitively, an estimated 13 million U.S. hypertensive treated people aged 60 years or older [11] would reduce pill intake as the SBP goal increase from 140 to 150 mmHg. Antihypertensive medication use may be associated with injurious falls in the elderly, a 30% to 40% increased risk compared with no antihypertensive medication use [12]. Moreover, the results of the ACCORD MIND [13] show that intensive management to a target SBP of less than 120 mm Hg and fibrate therapy in the context of LDL cholesterol level control are not effective in reducing cognitive decline in persons with poorly controlled type 2 diabetes at high risk for cardiovascular disease.

## The AACE diabetes guidelines

The 2013-AACE algorithm and consensus statement on diabetes [5] has been criticized with particular concerns about transparency, conflicts of interest, group composition, and the abundant use of personal judgment and experience instead of rigorous methodology [6]. Despite all these concerns, the ultimate feeling is that the major concern relates to the financial ties. Most panel members, including the chair, of the diabetes AACE guidelines had financial conflicts of interest [6], and many of the financial associations were with companies that sell diabetes medications that figure prominently in the algorithm and consensus statement.

## Producing guidelines: a hard job

Producing guidelines in the United States has become increasingly more complicated and contentious. The presence of conflicts of interest is a common source of controversy, with claims that recommendations are designed to fill the pockets of those who would profit from the interventions advocated [14]. All this leaves patients and clinicians perplexed and distrustful of guidelines, adding uncertainty to an imperfect science, as Medicine is. However, scientific U.S. guidelines deeply impact on the prescription patterns of thousands of clinicians, and on the health needs of millions of patients worldwide. All the story also demonstrates that even in topic areas (high cholesterol, hypertension, diabetes) with extensive amounts of data and published clinical trials, crucial evidence is still missing.

Approximately 2500 guidelines are operative in the U.S. (http://www.guideline.gov) with the aim to improve clinical guidance: about three-fifths were issued by a medical specialty society or a professional association. Even considering overlapping and homonymy, it seems reasonable to suppose that thousands of experts are involved in U.S. medical guidelines. But, who is an expert? A person who is very knowledgeable about or skilful in a particular area (Oxford Dictionaries); in the medical field, an expert is supposed to be a clinician with a solid scientific reputation and skilled experience in clinical care. Paradoxically, the scientific reputation of an expert may be based on clinical trials that are, for the most, sponsored by industry. Putting an expert among panel members for its capacity to attract media attention, should be nuanced by the high likelihood for financial conflicts of interest. In a recent cross-sectional study [15] evaluating 45 guidelines from Danish clinical specialty societies, 96% of guidelines had one or more authors with a conflict of interest (independent validation), but only 2% disclosed author conflicts of interest. Moreover, only 22% of guidelines described the methods used for guideline development and 24% graded the types of evidence.

## A suggested way out

Is there a honorable way out? For clinicians, who are called to operate a personal choice, based on clinical judgment, among the many guidelines release by reputed scientific associations; for experts, whose reputation may suffer from these reflections; and for the millions of people whose care may be affected. It may be hard to imagine that professional societies and scientific organizations can renounce to industry funding, especially in times, as the present, of financial shortage. Not surprisingly, important members of these societies or organizations may be the same experts called for writing guidelines. There is agreement that “clinicians and patients are most likely to pay attention to recommendations that are formulated by independent experts without funding from industry” [16]. A multidisciplinary panel with members who have no substantial financial and intellectual conflicts of interest is essential, also for attracting the attention of clinicians and patients to any clinical guideline. However, the search of experts without funding from industry may be unproductive, and freedom from any conflict (not only financial) can be difficult to ascertain. The American Cancer Society methodology aligns with the Institute of Medicine principles for trustworthy clinical guideline development, particularly by separating the processes of specialty input and evidence synthesis from writing of the actual guideline [17]. Separate careers for experts who collect evidence from persons who write the actual guidelines seems a good opportunity in order to attenuate the rumors associated with release of new guidelines, especially those that counter prior practice. Inclusion of multiple stakeholders during guideline development, evidence reviews that are fully available for scrutiny before a guideline is finalized, and refraining from turning the release of new or updated guidelines into media events [14] may also help.

## Competing interests

The authors take full responsibility for the content of this article. Dario Giugliano serves as guarantor. KE, AC, SG, and DG received consultancy fees, attended advisory boards or have held lectures for a number of pharmaceutical companies producing antidiabetic drugs.

## Authors’ contributions

KE and DG wrote the manuscript. All authors revised the article for important intellectual content. All authors read and approved the final manuscript.
